# Correction: *In vivo* study on the repair of rat Achilles tendon injury treated with non-thermal atmospheric-pressure helium microplasma jet

**DOI:** 10.1371/journal.pone.0316006

**Published:** 2024-12-13

**Authors:** Katsumasa Nakazawa, Hiromitsu Toyoda, Tomoya Manaka, Kumi Orita, Yoshihiro Hirakawa, Kosuke Saito, Ryosuke Iio, Akiyoshi Shimatani, Yoshitaka Ban, Hana Yao, Ryosuke Otsuki, Yamato Torii, Jun-Seok Oh, Tatsuru Shirafuji, Hiroaki Nakamura

The first author’s name is spelled incorrectly. The correct name is: Katsumasa Nakazawa. The correct citation is: Nakazawa K, Toyoda H, Manaka T, Orita K, Hirakawa Y, Saito K, et al. (2024) *In vivo* study on the repair of rat Achilles tendon injury treated with non-thermal atmospheric-pressure helium microplasma jet. PLOS ONE 19(5): e0301216. https://doi.org/10.1371/journal.pone.0301216.
