# Supplementary material for: In vivo study on the repair of rat Achilles tendon injury treated with non-thermal atmospheric-pressure helium microplasma jet
Source: PLoS One. 2024 May 14;19(5):e0301216. doi: 10.1371/journal.pone.0301216 (PMC11093389; doi:10.1371/journal.pone.0301216)
Supplement: Attachment — Submitted filename: renamed_6daee.docx [file pone.0301216.s002.docx]

**Reviewer #1: General comments:**

This is a very interesting manuscript, very well written and structured. The topic is of scientific infesters and with possible e future application in a clinical point of view.

**Specific comments:**

- The title should include some reference to this population, thus the rat as a model; Also this should be clearer in the abstract.

**RESPONSE:** Thank you for pointing this out. We have revised the title to “In vivo study on the repair of Rat Achilles tendon injury treated with non-thermal atmospheric-pressure helium microplasma jet ”.

- Line 79: After the objective, I suggest to add the hypothesis of this study;

**RESPONSE:** Thank you for this suggestion. We have revised the final paragraph as follows:

"We hypothesize that NTAPP irradiation of the Achilles tendon enhances the cell proliferative capacity and promote early Achilles tendon repair. ” in Lines 80–82.

- Line 146: When the authors refer to the histological evaluation by three authors. Do you mean blinded evaluation? Please specify this and how was the method of evaluation.

**RESPONSE:** Thank you for raising this point. The following text has been added to clarify this aspect: “Histological evaluation was performed by three investigators (*n* = 6) blinded to the treatment and number of weeks.” in Lines 148-149

- Results section: In this section there are some sentences that seems to discuss the results and should be on the discussion section and not here. For example: Line 247: "Thus, the appearance of type I and II ..."; Line 252: " This indicates that the collagen networks ..."; Line 263: " This clearly indicated early..."; Line 279: " These result show that NTAPP irradiation ...."Line 289: " thus plasma-treated improved...". All these are discussion or conclusions and should not be here.

**RESPONSE:** Thank you for pointing this out, we have moved this content from the Results to the Discussion.

- Discussion section: This section is very well written and I would only suggest to add some more bibliographic reference. For example: Line 303 when talking about scar tissue formation; line 321 when talking about achilles tendon maturation; line 352 when talking about the reports that have been published.

**RESPONSE:** Thank you for this suggestion. We have added the following references.

37) Schulze-Tanzil GG, Cáceres MD, Stange R, Wildemann B, Docheva D. Tendon healing: a concise review on cellular and molecular mechanisms with a particular focus on the Achilles tendon. Bone Jt Res. 2022;11: 561–574. doi:10.1302/2046-3758.118.BJR-2021-0576.R1

39) Thomopoulos S, Soslowsky LJ, Flanagan CL, Tun S, Keefer CC, Mastaw J, et al. The effect of fibrin clot on healing rat supraspinatus tendon defects. J Shoulder Elb Surg. 2002;11: 239–247. doi:10.1067/mse.2002.122228

40) Aro AA, Simões GF, Esquisatto MAM, Foglio MA, Carvalho JE, Oliveira ALR, et al. Arrabidaea chica extract improves gait recovery and changes collagen content during healing of the Achilles tendon. Injury. 2013;44: 884–892. doi:10.1016/j.injury.2012.08.055

41) Sharma P, Maffulli N. Tendon injury and tendinopathy: Healing and repair. J Bone Jt Surg. 2005;87: 187–202. doi:10.2106/JBJS.D.01850

42)Hou Y, Mao ZB, Wei XL, Lin L, Chen LX, Wang HJ, et al. The roles of TGF-β1 gene transfer on collagen formation during Achilles tendon healing. Biochem Biophys Res Commun. 2009;383: 235–239. doi:10.1016/j.bbrc.2009.03.159

- Paragraph 342-351: It is confusing I suggest to explain better.

**RESPONSE:** Thank you for this suggestion. The following text has been revised on Lines 354–361:

“NTAPP produces ROS and RNS, including nitrogen monoxide (NO) and nitrogen dioxide (NO_2_), by interacting with the surrounding atmosphere when gases are irradiated from the plasma jet [51]. Only recently has the role of ROS in the maintenance of normal biological functions become clear [52]. ROS activate intracellular signaling pathways, resulting in the production of soluble factors involved in cell growth and proliferation [53,54]. According to Brun et al., He-generated plasma treatment induces the proliferation and migration of primary human fibroblast-like cells, primarily through intracellular ROS formation [15]. Accordingly, we speculate that the RONS generated through the plasma jet may promote tendon regeneration.”

- Paragraph 360- 365: Once again the hypothesis should be first mentioned after the objective and then I also suggest to re-write this part for better understanding.

**RESPONSE:** Thank you for this comment. The following text has been revsied on Lines 370–372:

"Therefore, NTAPP irradiation might induce fibroblast proliferation and migration, increase the production of cytokines involved in tendon repair, and improve Achilles tendon healing. However, these hypotheses require further verification.”

- Line 372: Replace "here" for something more specific as " in this study" or " with this population" or " in this case", etc.

**RESPONSE:** Thank you for these suggestions. The following text has been revised on Lines 383–384:

“In conclusion, this study shows early tissue repair and the conversion of collagen type III to type I were accelerated by the NTAPP irradiation of the Achilles tendons.”

- In the last paragraph I suggest to make a point on how this could be important for future application in the clinical setting.

**RESPONSE:** Thank you for this suggestion. We have added a brief summary of potential future clinical applications in the final paragraph as follows:

“The clinical application of NTAPP's restorative effect on Achilles tendon injuries is expected to shorten the bracing period and enable an earlier return to sports. It may also prove effective for the treatment of other tendon injuries, and is expected to be explored further in this context in the future.” (Lines 388–390)

**Reviewer #2:** In their paper “In vivo study on the repair of Achilles tendon injury treated with non-thermal atmospheric-pressure helium microplasma jet” Nakazawa and co-worker have tenotomized rats. Afterwards they have resuturerd the Achilles’tendon and applied helium plasma to one group. The healing success was investigated using histology, biomechanics and XPS. The set-up appeared to be sound. There appear to be an effect of the treatment, which is significantly measurable. However even I value Gundula Schulze-Tanzil and her group greatly, I do not agree to put all histomorphometric parameters into one score. Especially, when experiencing different issues. On the one hand there is enchondral ossification (due to tendon degeneration) which is detectable by alzian blue staining. However, this staining will disappear over time when the degenerated tendon goes into ossification. On the other hand there is scar formation, which is found by several other parameters (like cellularity). Putting these two things in the same basket is not the same in my point of view.

**RESPONSE:** Thank you very much for your suggestions. We have conducted a statistical review again for each item. Regarding the text, we have added the following on Lines 239–242 and added Table 2:

“The item-by-item comparison revealed that there was no significant difference between items at 2 weeks. However, at 4 weeks, significant differences were observed in cell alignment, cell nucleus morphology, and vascularization in the defect area. At 6 weeks, a significant difference occurrent in the transition from defect to normal tissue."

We have also included added a description of endochondral ossification on Lines 320–322 as follows:

“In addition, Alcian blue staining revealed cartilage formation starting 2 weeks post-operatively. These cartilage masses reportedly calcify after 4 to 6 weeks and are thought to be caused by endochondral ossification [44].”

The quantitative analysis of collagen I is unfortunately completely unacceptable in this form. Picrosirius staining with polarization microscopy is not a way to quantify the collagen I content in tendons. The color shift is an indicator for fibril thickness but not for a change in protein. Collagen type I can be of every color in picro-sirus staining depending on the collagen maturation stage and collagen type V content. Speaking of collagen type V- please reinvestigate line 242-248 because there are many things which are not right like the role of collagen V. Also there is a gradient of collagen I to collagen III dependent on the tissue and stage. Skin has quite a big portion of collagen type I besides collagen type III. In tendon it is inversed. The minor fibrillar collagens are not correctly mentioned too.

**RESPONSE:** Thank you very much for your suggestion. The role of type V collagen and the contents regarding Picrosirius red staining were added to Lines 327–332 as follows:

“Normal Achilles tendon dry weight is composed primarily of collagen type I (90%) [45,46]. However, it also contains type III collagen, present in the fibrocartilage of tendons and tendons, as well as type V collagen, which forms the nuclei type I collagen protofibrils and participates in fiber diameter regulation [47]. Picrosirius red staining detects and quantitatively evaluates collagen in normal and overlying tissue sections and can effectively identify collagen type I and collagen type III in the musculoskeletal system, liver, gastrointestinal tract, skin, and myocardium [48].”

As you noted, there was a possibility of type V collagen involvement. Accordingly we have included the following as a study limitation:

“Fourth, the quantitative evaluation of type I collagen content via picrosirius red staining may be affected by the maturation stage of collagen and the type V collagen content, which must be considered when interpreting the results." (Lines 378–380)

Please give us a graphical representation of the results of the biomechanics and picro-sirius quantification, there are better to internalize than the numbers inside the text.

The discussion is not sound since you have overinterpreted the picro-sirius data. To draw these conclusions you need other methods, like mass spectrometry or antibody driven detection methods (immune fluorescence or western blotting).

**RESPONSE:** We have addressed this comment as a study limitation on Lines 380–382 as follows:

“Hence, mass spectrometry analyses or antibody detection methods (immunofluorescence or western blotting) are warranted to address these possibilities.”

We have also added the biomechanics and picro-sirius quantification results to Figures 10 and 11.
