# Supplementary material for: In vivo study on the repair of rat Achilles tendon injury treated with non-thermal atmospheric-pressure helium microplasma jet
Source: PLoS One. 2024 May 14;19(5):e0301216. doi: 10.1371/journal.pone.0301216 (PMC11093389; doi:10.1371/journal.pone.0301216)
Supplement: Attachment — Submitted filename: renamed_e4f30.docx [file pone.0301216.s003.docx]

**Reviewer #1: General comments:**

This is a very interesting manuscript, very well written and structured. The topic is of scientific infesters and with possible e future application in a clinical point of view.

**Specific comments:**

- The title should include some reference to this population, thus the rat as a model; Also this should be clearer in the abstract.

**RESPONSE:** Thank you for pointing this out. We have revised the title to “In vivo study on the repair of Rat Achilles tendon injury treated with non-thermal atmospheric-pressure helium microplasma jet ”.

Reviewer #1: This manuscript is well structured and debates an important theme for the clinical practice. Although it is performed in rats, it has the potential to be translated for dogs and cats. I think this study should be continued and try to apply in dogs and cats.

The authors coped with every proposed alteration, and thus it is acceptable for publication.

**RESPONSE:** Thank you very much for your evaluation. We would like to study the effects of low-temperature atmospheric pressure plasma using large animals in the future.

Reviewer #2: I still think that the authors should add an additional way to quantify the collagen content. The authors have prepared the histological slides and can quantify collagen IHC.

**RESPONSE:** Thank you for pointing this out. We have now added Immunohistochemical testing for collagen type Ⅰ and Ⅲ. Collagen type Ⅰ was increased in the plasma treatment group during the entire period, and collagen Ⅲ was decreased at 2 and early 4 weeks.

The above results are noted in the text as follows;

Line 167-179

“Immunohistochemical testing

Paraffin-embedded sections were deparaffinized using xylene and dehydrated through graded alcohols. Slides were pretreated with citrate buffer (Target Retrieval Solution [S1699], 10×; DAKO Japan, Tokyo, Japan) in phosphate-buffered saline solution (PBS) for 20 minutes at 90°C for optimal antigen retrieval. Endogenous peroxidases were quenched using 1.0% hydrogen peroxidase in methanol for 30 minutes at room temperature. Slides were then rinsed with PBS and incubated with 10% goat serum for 30 minutes at room temperature. Subsequently, specimens were incubated with rabbit primary antibodies (type I collagen, 1:100 dilution; Abcam, ab34710 and type III collagen, 1:100 dilution; Abcam, ab6310) at 4°C overnight. After extensive washing with PBS, slides were incubated with a peroxidase-labeled antibody (Histofine Simple Stain; Nichirei Biosciences, Tokyo, Japan) for 30 minutes at room temperature. After extensive washing with PBS, the immunoreaction was visualized by incubating the sections for 3 minutes in 3,3′-diaminobenzidine (Histofine Simple DAB solution; Nichirei Biosciences).”

Line 283-306

**“Quantitative analysis of the type I and III collagen content via immunohistochemistry testing**

Type I collagen expression was observed in the plasma-treated group from 2 weeks and became stronger with time, showing stronger staining compared to the untreated control group during the entire period (Fig 11 A-F). Type III collagen expression was stronger in the untreated control group than in the plasma-treated group at 2 and 4 weeks (Fig 12 A, B, D, and E), but the staining was similar at 6 weeks (Fig 12 C and F). Expression decreased over time in both groups. In quantitative analysis, type I collagen expression was significantly higher in the plasma-treated group at 2, 4, and 6 weeks. (p = 0.010, p = 0.025, and p = 0.025, respectively). Type III collagen expression was significantly lower in the plasma-treated group at 2 and 4 weeks (p = 0.010 and p = 0.037), but there was no significant difference between the two groups at 6 weeks. (p = 0.262) (Fig 13)

Fig 11. Immunohistochemical testing against type I collagen results. (A–C) Tissue after He microplasma jet treatment at 2, 4, and 6 weeks post-surgery, respectively. (D–F) Untreated control tissues at 2, 4, and 6 weeks post-surgery, respectively. Scale bar, 200 µm (originally 100 µm).

Fig 12. Immunohistochemical testing against type　Ⅲ collagen results. (A–C) Tissue after He microplasma jet treatment at 2, 4, and 6 weeks post-surgery, respectively. (D–F) Untreated control tissues at 2, 4, and 6 weeks post-surgery, respectively. Scale bar, 200 µm (originally 100 µm).

Fig 13. Quantitative analysis of the type I and Ⅲ collagen content via Immunohistochemistry testing

Type I collagen expression in He microplasma jet-treated group was higher than that in untreated control group in all time point. (a: p = 0.010, b: p = 0.025, c: p = 0.025) Type III collagen expression was significantly lower in the He microplasma jet-treated group at 2 and 4 weeks. (a: p = 0.010, b: p = 0.037)”

We have also added the immunohistochemical testing results to Figures 11, 12 and 13.

Fig. 13 is changed to 14 on Line 294-306.

Discussion also edits as follows.

Line 338-339

“Immunochemical staining results also showed more type 1 collagen in the plasma-treated group and less type 3 collagen in the plasma-treated group at 2 and 4 weeks.”

Line 375-378

“Here, the type I collagen content also resulted in higher brightness in Picrosirius red staining and the expression of type I collagen in immunochemical stainning at 2, 4, and 6 weeks post-surgery. The expression of type III collagen was also decreased in the plasma treatment group at 2 and 4 weeks.”
